# Ideational factors associated with net care behaviour: a multi-country analysis

**DOI:** 10.1186/s12936-022-04053-5

**Published:** 2022-02-17

**Authors:** E. ‘Kuor Kumoji, Grace N. Awantang, Michael Toso, Diarra Kamara, Thérèse Bleu, Wani Lahai, Musa Sillah-Kanu, Abdul Dosso, Dorothy Achu, Stella Babalola

**Affiliations:** 1 Johns Hopkins Center for Communication Programs, 111 Market Place, Suite 310, Baltimore, MD 21202 USA; 2 Programme National de Lutte Contre Le Paludisme, Abidjan, Côte d’Ivoire; 3 National Malaria Control Program, Ministry of Health and Sanitation, Freetown, Sierra Leone; 4 Programme National de Lutte Contre Le Paludisme Au Cameroun, Yaounde, Cameroon; 5 Johns Hopkins Bloomberg School of Public Health, Baltimore, MD USA

**Keywords:** Malaria, Insecticide-treated net, Bed nets, Net care, Ideation, Social and behaviour change communication, Cameroon, Côte d’Ivoire, Sierra Leone

## Abstract

**Background:**

Malaria is endemic to sub-Saharan African countries. Mass and routine distribution, promotion, and use of ITNs are critical components of malaria prevention programmes. Correct and consistent use of insecticide-treated mosquito nets (ITN) is an effective strategy for malaria prevention. To extend bed-net lifespan, the World Health Organization recommends folding or tying up ITNs when they are not in use. This study analyses factors associated with net care practices in three African countries.

**Methods:**

Researchers collected household data nationwide in Côte d’Ivoire, from the North and Far North regions of Cameroon, and from Port Loko and Bo districts in Sierra Leone, between 2018 and 2019. The dependent variable was respondents reporting that they fold or tie up their nets. The study adjusted for selected sociodemographic, ideational (psychosocial), and household variables using multilevel models. The analysis was limited to women of reproductive age and their male spouses/partners from households with at least one ITN: 2,940 respondents in Cameroon, 6,105 in Côte d’Ivoire, and 2,730 in Sierra Leone.

**Results:**

Among respondents, 50.2% in Cameroon, 52.0% in Côte d’Ivoire and 75.6% in Sierra Leone reported folding or tying up their net when it was not in use. In all three countries, the data showed significant clustering at both household and community levels, indicating the influence of factors operating at these levels on net-care behaviour. The odds of reporting the behaviour varied significantly by geographic unit in each country. Consistent use of nets was strongly correlated with net-care behaviour. Furthermore, five ideational variables were positively associated with the outcome behaviour in all three countries: positive attitude towards net care, perceived susceptibility for malaria, response-efficacy of ITNs, perceived self-efficacy for net use, and the perception that net use was a community norm. Additional significant ideational variables included positive attitudes towards net use (Cameroon and Côte d’Ivoire), perceived severity of malaria (Côte d’Ivoire), and interpersonal communication about malaria (Côte d’Ivoire).

**Conclusions:**

The study identified ideational variables associated with recommended net-care practice. Programme efforts designed to promote net-care practices and extend average lifespan of ITNs may be more effective if they emphasize positive attitudes towards net care, perceived susceptibility of malaria infection, response-efficacy of ITNs, perceived self-efficacy for net use, and promote net-care behaviour as a positive community norm.

**Supplementary Information:**

The online version contains supplementary material available at 10.1186/s12936-022-04053-5.

## Background

For the majority of households in sub-Saharan Africa, insecticide-treated nets (ITNs) are effective tools for preventing malaria. While routine distribution of ITNs through channels such as antenatal care are becoming more common, most families in this region receive ITNs through mass distributions that take place about every three years [1]. While the physical integrity and bio-efficacy of ITNs last for approximately three years under normal use conditions [2], and in many countries in sub-Saharan Africa mass distributions are timed accordingly [3], access to ITNs usually drops between distribution campaigns [3, 4].

Recent studies in several countries indicate that the physical integrity of ITNs tends to be compromised (e.g., holes, tearing) before degradation of insecticidal activity [5–10]. This is a concern for malaria prevention because when nets have holes, protection from mosquitoes is compromised and damaged nets are more likely to be discarded, repurposed or no longer used [11–17].

World Health Organization (WHO) guidelines for monitoring the durability of ITNs recommend measuring net survivorship (i.e., proportion of distributed nets still available for use as intended in the households to which they were given, after one, two, three or more years), fabric integrity (i.e., the number, location and size of holes in each net), and insecticidal activity (i.e., the degree of knock-down, mortality or inhibition of blood-feeding induced in susceptible mosquitoes, as determined by standard WHO test procedures and criteria) associated with nets [18]. A decade of ITN durability monitoring data indicates that the frequency of washing, methods of airing and drying, repair, and folding or tying ITNs up when they are not in use, are all net-care behaviours in the household that potentially preserve or reduce the fabric integrity and/or insecticidal activity of ITNs [5, 6, 8–10, 14–19]. In addition, WHO has listed the presence of rodents, use of nets by children, and fire as factors that are known to affect ITN durability [18].

Net-care practices are commonly studied as a factor that affects ITN durability; however, in many of these research studies, net care was not the primary outcome of interest but rather a factor correlated with the physical condition of the net [5, 22]. Studies show that tying or folding ITNs when they are not in use is effective in extending the useful lifespan of nets [20, 21]. For example, results from a study in Nigeria showed that nets that were tied up were almost three times as likely to be in serviceable condition compared to nets that were not [20]. Another study in western Kenya showed a positive link between a composite variable representing the practice of seven net-care behaviours (including tying or folding up nets when not in use) and a net’s condition after adjustment for net age [21].

Behaviour change messages have also been correlated with proper net-care behaviours. A study from Uganda found that the proportion of campaign nets hanging was significantly higher in the district with radio broadcasts and community events relative to the comparison district [23]. A few studies have linked exposure to social and behaviour change (SBC) interventions with net durability or condition, possibly because exposure to those messages led to increased proper net-care practices [20, 23, 24]. For example, in Nigeria [20], a study found a strong positive link between exposure to SBC messages and positive attitude towards net care and repair. The same study also found that a positive attitude towards net care was one of the most significant predictors of longer net lifespan. One study found that a child under the age of five years using the net and sharing the dwelling unit with domestic animals increased the odds of proper net storage [21].

Research on health communication for behaviour change positions ideation or psychosocial determinants of behaviour as a key intermediate construct between contextual factors and behaviour [25]. The ideation model describes constructs known to be proximal determinants of intentions to act and actual behaviour, including cognitive and emotional factors, as well as elements of social interactions. Ideational determinants such as self-efficacy to obtain enough nets [26–28], that is, one net for every two people in the household, and perceived severity of malaria [26, 29] have been linked to the practice of net use. Few studies have described the psychosocial or ideational determinants of net care. Evidence on the ideational determinants of proper net care in general, and tying/folding up nets in particular, is limited to qualitative and descriptive quantitative studies conducted in Nigeria [30], Senegal [31], Tanzania [32], Ethiopia [33], and western Kenya [21]. Qualitative studies identified the desire to keep ITNs away from children [31], social pressures to keep ITNs clean [30], desired protection from mosquitoes [32], and perceived benefits of having a net in good condition [34] as reasons for tying or folding ITNs up when they are not in use. These studies also identified time required to tie and fold up multiple nets in the household as a barrier to net care behaviours [30, 31]. A quantitative study in western Kenya found that knowledge of appropriate net-care practices increased the odds of tying or folding ITNs up [21].

The timing of mass ITN distributions preceding this study’s data collection is noteworthy because net-care messages may have accompanied distributions. The timing of the most recent mass net distribution activity conducted in the study areas varied among and within countries relative to when data collection occurred. In Cameroon, a mass distribution campaign coincided with data collection in the North region but preceded data collection in the Far North region by three years (2016). In Côte d’Ivoire, the most recent mass net distribution was launched about one year before data collection began in October 2017, whereas the Malaria Behaviour Survey (MBS) data collection took place between 2 September and 11 November, 2018. The 2017 Sierra Leone mass net distribution was launched in June, long before MBS data collection took place between September and October 2019.

This is the first multi-country comparison of the ideational correlates of net care behaviours (tying or folding ITNs up when they are not in use) and the first to examine consistent net use as a correlate of these behaviours. A better understanding of ideational determinants of tying or folding ITNs up will help malaria control and elimination programmes design SBC interventions that increase the average effective ITN lifespan and may contribute to better sustained ITN coverage between net distribution campaigns.

## Methods

### Study population and setting

This study analysed data from three cross-sectional malaria behaviour surveys in Cameroon, Côte d’Ivoire and Sierra Leone, implemented as community-based household surveys. The surveys were designed to estimate the prevalence of malaria-related prevention, care-seeking and treatment behaviours and their relationship with ideational factors, including attitudes towards caring for and using nets. The three surveys used nearly identical data collection tools and sampling protocols. The tools assessed malaria prevention strategies adopted by each country: ITN use, intermittent preventive treatment of malaria in pregnancy using sulfadoxine-pyrimethamine (IPTp-SP), case management, seasonal malaria chemoprevention (SMC)/intermittent preventive treatment of malaria in infants (IPTi), and indoor residual spraying (IRS) modules were included in the Sierra Leone and Côte d’Ivoire tools, while Cameroon omitted the IRS module. There were minor revisions to wording of questions and response options for contextual relevance.

Decisions about the scope of the survey were made in consultation with the Ministry of Health and National Malaria Control Programme in each country. In Côte d’Ivoire and Sierra Leone, the Government Offices of Statistics helped to select the study enumeration areas in urban and rural areas with probability proportional to size sampling and supplied enumeration area sketch maps. In Cameroon, the National Institute of Cartography provided this service. The survey was national in Côte d’Ivoire but conducted in two districts in Sierra Leone and two regions in Cameroon. In all three countries, a multi-stage, stratified sampling approach was used; this approach started with stratifying the study locations (zones in Côte d’Ivoire, districts in Sierra Leone, regions in Cameroon) into urban and rural areas. Subsequently, a number of enumeration areas were selected within each stratum using probability proportional to size techniques. Field workers visited each selected enumeration area, listed all households in the enumeration area and randomly selected between 21 and 23 eligible households for inclusion in the survey. In each selected household, all women of reproductive age were targeted for interview; in one third of selected households, the spouse of one of the recruited women was targeted for interview. Detailed information on MBS methods is available at https://malariabehaviorsurvey.org/about.

The survey in Côte d’Ivoire was nationally representative while the other two focused on specific locations in Sierra Leone (Bo and Port Loko districts) and Cameroon (North and Far North regions). The study population included the head of each household or their representative, women of reproductive age within each household, and for a portion of participating women, a male partner/husband was also interviewed.

### Sample size

The sample size was calculated for the three cross-sectional surveys to provide estimates of certain indicators with a specified degree of certainty and to produce programmatically useful data. Sample sizes for each country were calculated based on the prevalence of three key indicators using the formula below:

$$n = d \times \frac{{Z_{{1 - \frac{\alpha }{2}}}^{2} \times p(1 - p)}}{{\delta^{2} \times R_{h} \times R_{i} }}$$

where:

- n is the required sample of individuals (e.g., women)
- Z is the Z value corresponding to the desired 95% confidence level
- d is the design effect due to departure from simple random sampling (2.0 in Sierra Leone, 2.5 in Cameroon, and 3.0 in Côte d’Ivoire).
- p is the estimated (expected) outcome of interest. The same three outcome indicators were used in each country:
  - proportion of women of reproductive age with positive attitudes towards consistent use of mosquito nets
  - the proportion of women of reproductive age that slept under a net on the night before the survey
  - the proportion of children under five years of age that had fever in the last two weeks
    For outcomes that were not available in the publicly available data sets (e.g. Malaria Indicator Survey (MIS) or the Multiple Indicator Cluster Survey (MICS), a proportion of 50% (p = 0.5) was assumed for maximum variability and consequently maximum sample size.
- δ is the desired margin of error (δ = 6% in Cameroon, 5% in Côte d’Ivoire and 5% in Sierra Leone)
- R_h_ is the response rate for households; a response rate of 90% was assumed in all three countries.
- R_i_ is the response rate for women in selected households; a response rate of 96% was assumed in all three countries.

The number of households that would need to be approached for each sub-national region: Port Loko and Bo (Sierra Leone), North zone, Central zone, South zone, and Abidjan (Côte d’Ivoire), and Far North and North regions of Cameroon was calculated for all three indicators. In each country, the maximum number of households that would need to be approached was selected from each indicator estimate as a target for data collection teams in that sampling area. The sampling strategy for the MBS is described in further detail at https://malariabehaviorsurvey.org/methods/

### Data collection

Trained teams implemented the same sampling procedures to collect survey data during the rainy season in each country. Data collection took place between September and October 2019 in Cameroon, between September and November 2018 in Côte d’Ivoire, and between September and October 2019 in Sierra Leone. Teams visited each sampled enumeration area and listed households to facilitate sample selection and data collection. Systematic random sampling was used to select households within each enumeration area. Interviewers used mobile devices to collect responses from household heads or their representatives in households where at least one woman of reproductive age lived. Household heads provided information about the household’s socio-economic status, the number of household members, and the number of ITNs in the household. In each selected household, data collectors interviewed all eligible women of reproductive age. In every third household, one participating woman’s male partner/spouse was also interviewed. More women than men were sampled to better capture malaria prevention behaviours usually performed by women such as accessing antenatal care (ANC) and IPTP-SP, accepting IPTi, and care-seeking for fever in young children. By interviewing men in one-third of the households, the survey also was able to better understand men’s views as key household decision-makers. Interviewers asked each adult about how they cared for the nets in the house as well as their attitudes and other perceptions related to net care, net use, and malaria in general (see Table 1).

**Table 1:** Description of independent variables examined in analysis

Background variables	Description
Gender	Male or female, as established during recruitment
Current age	Age in years
Education level	Highest level of formal school attended, if any
Exposure to messages in last six months	Whether respondent had seen or heard any messages about malaria in the past six months
Household size	Sum of household listing from household questionnaire Number of individuals, of any age, living in the household in question
Number of nets in the household	Sum of net listing from household questionnaire, total number of mosquito nets in the household
Household wealth quintile	Categorical variable with five potential values representing poorest to wealthiest household, a relative measure of wealth constructed using primary component analysis from questions related to characteristics of the interviewee’s dwelling (e.g., roof, wall, ceiling materials, access to utilities, and ownership of electronic devices, land, cattle)
Urban vs. rural setting	Refers to the relative population density of the enumeration cluster as determined by the institution who provided the enumeration maps for survey sampling
Region/ district/zone	Largest subnational administrative unit from which household were sampled
Ideational variables	
Positive net care attitudes*	Each respondent’s index score was summed across the statements. The resulting score for the two statements varied from -2 to + 2. A binary variable was created by classifying those with a negative or zero score as (0) not having positive net care attitudes. Those with a positive score (above zero, 1) were considered to have positive net care attitudes
Positive attitudes towards net use*	Each respondent’s index score was summed across the six statements. A binary variable was created by classifying those with a negative or zero score as (0) not having positive attitudes towards net use. Those with a positive score (above zero, 1) were considered to have positive attitudes towards net use
Severity of malaria *	Each respondent’s index score was summed across the four statements. A binary variable was created by classifying those with a negative or zero score as (0) not perceiving the consequences of malaria infection as severe (i.e., perceived severity of malaria). Those with a positive score (above zero, 1) were considered to perceive the consequence of malaria as severe
Perceived susceptibility to malaria*	Each respondent’s index score was summed across the four statements. A binary variable was created by classifying those with a negative or zero score as (0) not perceiving themselves as susceptible to malaria infection. Those with a positive score (above zero, 1) were considered to perceive themselves as susceptible to malaria infection
Discussed malaria with spouse, friends or relations in the last six months	Responses to the two questions were collapsed into a binary variable such that a respondent who answered “Yes” to both questions was assigned a value of (1) and a respondent who answered “No” to at least one question was assigned a value of (0)
Perceived response efficacy of nets*	Each respondent’s index score was summed across the three statements. A binary variable was created by classifying those with a negative or zero score as (0) not perceiving nets as an effective way to prevent malaria. Those with a positive score (above zero, 1) were considered to perceive nets as an effective way to prevent malaria
Perceived self-efficacy for net use*	Unlike the other Likert statements, respondents were asked to indicate if they were confident in their ability to perform a particular action by selecting “could,” or “could not.” Each respondent received a score for their response: (− 1) could not, (0) don’t know/not sure/missing, and (1) could. Each respondent’s index score was summed across the four statements. A binary variable was created by classifying those with a negative or zero score as (0) not being confident that they could use a net consistently. Those with a positive score (above zero, 1) were considered to be confident that they could use a net consistently
Perceived net use as the norm in one’s community	Responses to this question were re-coded such that respondents who indicated one of the first three options were coded (1) and respondents who indicated one of the last two options, or for whom the response was missing, were coded (0)

^*^Variable was created based on scoring and dichotomizing the sum of scored Likert scale questions

Field work was conducted by local data collectors in each country who were trained on ethical considerations for human subjects’ research by the study principal investigator, including informed consent, the study protocol and data collection tools. Data collectors used mobile devices and open data kit-based software (Survey-To-Go in Cameroon and Côte d’Ivoire; Survey CTO in Sierra Leone) to collect data. The study principal investigator for each country supervised field work and monitored data quality. The data were submitted to a secure online data management system on a daily basis.

The total population of households from which data were collected was 8,566 in Côte d’Ivoire, 3836 in Sierra Leone, and 4514 in Cameroon. As this paper focuses on ideational factors associated with net care, the analysis includes only the sub-set of households that had at least one ITN: 4264 households in Côte d’Ivoire, 1892 households in Cameroon, and 2077 households in Sierra Leone. This corresponded to a final sample of 6164 adults (1378 men and 4786 women) in Côte d’Ivoire, 2,995 adults (640 men and 2355 women) in Cameroon, and 2730 adults (456 men and 2274 women) in Sierra Leone. Refusal rates were 2% or less for household and individual participants in each country.

### Human subjects

Each study was approved by the Johns Hopkins School of Public Health Institutional Review Board as well as the appropriate national research ethics authority in each country prior to contact with study participants, namely: the National Ethics and Research Committee in Côte d’Ivoire, the Office of the Sierra Leone Ethics and Scientific Review Committee in Sierra Leone, and the National Human Health Research Ethics Committee in Cameroon. Data collection teams received training on ethical guidelines and the rights of human research participants. As part of the informed consent process, trained data collectors verbally explained the purpose of the survey, the types of questions that would be asked, the potential risks associated with participating in the survey, and the actions the study team will take to protect the privacy of the participants and keep the data confidential. In addition, data collectors informed participants that they did not have to participate in the study, they could decide at any point to discontinue their interview, and they did not need to answer any questions they did not want to. Written consent/assent was obtained in each of the three countries. Recruitment, consent and assent procedures were designed and implemented to ensure voluntary participation.

### Data analysis

#### Dependent variable

Researchers asked respondents what they did, if anything, to prevent nets from tearing or getting holes in them. The binary dependent variable reflects whether or not the respondent reported folding up or tying their net when not in use, in response to this question.

#### Independent variables

Analysis focused on the association between the dependent variable and household, sociodemographic, and ideational variables. Sociodemographic characteristics of the respondents included their gender, age and exposure to messages related to malaria in the last six months. Household demographic characteristics included household wealth quintile, household size, number of nets in the household, place of residence (i.e., rural or urban), and region or district of the country. Ideation variables included attitudes towards net use, net-care attitudes, perceived severity of malaria, perceived susceptibility to malaria, malaria-related discussion, perceived self- and response-efficacy, and perceptions of net use as a community norm. Lastly, the association between the dependent variable and whether a respondent reportedly slept under a net every night was also examined (see Table 1).

The questions linked with each ideational factor were converted into independent binary variables for each ideational element. This was done in a similar fashion as the procedure described in the 2017 Roll Back Malaria Partnership Malaria SBC Communication Indicator Reference Guide [35]. The questionnaire allowed for the creation of the binary ideational variables (see Additional file 1).

Most of the ideational variables were measured by asking respondents to indicate agreement or disagreement with Likert statements. Each respondent received a score for each question based on their response: (− 1) disagree, (0) don’t know/not sure/missing, and (+ 1) agree. If disagreement with the statement corresponded to a favourable response, the scoring for that particular statement was reversed. Thus, the respondent received a positive score for disagreeing (+ 1) with the statement. Second, an index score was calculated to reflect how each individual responded to the overall set of questions for an ideational factor. Each respondent’s index score was summed across the statements or questions. For example, three Likert scale statements were used to measure the perceived efficacy of nets to prevent malaria, the resulting index score would be integer values ranging from (− 3) to (+ 3). Third, based on their index score, each respondent was classified as having expressed a favourable ideational construct or not. A binary variable was created by classifying respondents with a zero or negative index score as *not*, for example, believing nets effectively prevent malaria (low response efficacy related to net use). Conversely, those with a positive score (above zero, 1) were considered to have high response efficacy and believe that nets were effective in preventing malaria.

### Analytic methods

All data were cleaned and analysed using Stata 16.0. The data were analysed and compared by country rather than in aggregate due to differences in the scale and representativeness of the data between countries. Descriptive, bivariate and multivariate analysis were conducted on the survey data. Geographic and sociodemographic variables that are known to influence behavioural outcomes were included in the final models as well as variables that were significantly associated with the outcome. The specific descriptive and multivariate results shown reflect the key results of our study. Adjusted Wald tests were used to compare the proportions of different groups of respondents who reported they tied or folded up their net when not in use with different background characteristics (e.g., region, wealth quintile, gender). Multilevel logistic regression was used to account for the similarities among respondent responses within each household and the similarities among households within each sampled cluster. Intra-class cluster coefficients (ICC) were calculated to assess the need to incorporate random effects in each model.

## Results

### Description of the sample

Demographic description of the sample for each country is shown in Table 2. In Cameroon, the largest proportion of survey respondents were between 25 and 34 years old (35.1%) while the bulk of the remaining respondents were slightly younger (15–24 years, 21.8%) or slightly older (35–44 years, 27.7%). About half of the respondents were Muslim (52.4%) while slightly fewer were Christian (44.3%). Almost two-thirds of respondents had no or little formal education (62.2%) while almost a third had completed primary school. Just over two-thirds (67.3%) of the respondents live in rural areas while the rest lived in urban areas.

**Table 2:** Demographic characteristics of the sample by country

	Côte d’Ivoire		Cameroon		Sierra Leone	
Background characteristics	Weighted per cent	Unweighted per cent	Weighted per cent	Unweighted per cent	Weighted per cent	Unweighted per cent
Gender						
Male	45.5	21.6	44.2	20.9	47.8	16.4
Female	54.5	78.5	55.9	79.1	52.2	83.7
Age category						
15–24 years	19.1	24.2	21.8	28.9	22.9	34.5
25–34 years	32.2	35.2	35.1	35.8	26.2	31.3
35–44 years	28.9	26.4	27.7	24.1	28.8	24.5
45 + years	19.8	14.2	15.4	11.2	22.1	9.7
Religion						
Christian	50.2	53.1	44.3	47.5	19.9	16.2
Muslim	41.1	38.2	52.4	49.9	80.1	83.8
Others	8.8	8.7	3.3	2.7		
Educational level						
No formal education or some primary	33.8	48.5	62.2	63.4	45.4	49.0
Completed primary	22.7	23.9	31.1	29.0	18.0	21.8
Completed secondary or higher	43.5	35.5	6.7	7.7	36.6	29.3
**Residence**						
Rural	37.6	46.0	67.3	51.8	70.2	80.63
Urban	64.4	53.9	32.7	48.2	29.8	19.37
District/Region/Zone						
Côte d’Ivoire						
North	15.5	19.3	–	–	–	–
Central	29.7	32.7	–	–	–	–
South	28.5	31.7	–	–	–	–
Abidjan	26.3	16.3	–	–	–	–
Cameroon						
North	–	–	44.6	52.8	–	–
Far North	–	–	55.4	47.2	–	–
Sierra Leone						
Bo	–	–	–	–	54.8	39.7
Port Loko	–	–	–	–	45.2	60.4

In Côte d’Ivoire, the weighted results indicate that 51.1% of the study population were between 25 and 44 years old while about one fifth were less than 25 years old. Christians made up about half of the population while Muslims were 41.1%. About one-third of the population were without any formal education whereas more than two-fifths (43.5%) had secondary education or higher. Urban residents made up about two-thirds of the study population. The distribution by zone showed that there were respondents from the Centre and South zones compared to the North zone.

In Sierra Leone, about half (49.1%) of the study population were under 35 years old, and almost a quarter (23%) of them were under 25 years old. The majority was Muslim (80.1%) and resided in rural areas (70.2%). Nearly half (45.4%) of participants had no or incomplete primary education, however 37% had completed secondary or higher education. There were slightly more participants from Bo district compared to Port Loko district.

In summary, more participants from Cameroon and Sierra Leone resided in rural areas and had no or incomplete primary education, compared to Côte d’Ivoire. The study population also was younger and predominantly Muslim in Cameroon and Sierra Leone, compared to those from Côte d’Ivoire.

### Cameroon

#### Prevalence and variations in net-care behaviour

Almost two-thirds (72.6%) of survey respondents in households with a net said they used a net consistently and roughly half (52.5%) of the same sample mentioned that they rolled up or tied up nets when they were not in use. These proportions varied significantly by region and gender. Respondents in the North region (64.4%) were less likely to report using a net every night than those in the Far North (79.5%) to practice this net-care behaviour (p < 0.001). Conversely, respondents in the North region (59.4%) were more likely than those in the Far North (44.2%) to practice this net-care behaviour (p < 0.001). Women were slightly more likely (53.5%) than men (48.6%) to report folding up or tying up nets when they were not in use (p < 0.05). Living in an urban or rural setting was not associated with practicing this behaviour; nor was a respondent’s household’s wealth quintile.

#### Multivariable analysis

The adjusted, multivariable, multilevel logistic regression (Table 3) indicated that several variables were significantly correlated with folding or tying up a net when not in use. The odds of practicing this behaviour were elevated among respondents who had positive attitudes towards net care, positive attitudes toward net use, who perceived themselves to be susceptible to malaria infection, who were confident in their ability to use a net consistently, and who perceived net use as a community norm. The odds of practicing this behaviour among respondents with positive attitudes towards net use (adjusted odds ratio (AOR) = 2.08, p < 0.001) was doubled compared to similar respondents without this positive ideational characteristic. The same was true among respondents with self-efficacy to use nets consistently (AOR = 2.17, p < 0.001) and those who perceived net use to be a norm (AOR = 2.02, p < 0.001). Perceived response efficacy of nets was negatively correlated with folding or tying nets up when not in use (AOR = 0.58, p < 0.001). The odds of folding or typing up a net was over twice as likely among respondents who reported that they slept under a net every night compared to similar respondents who slept under a net less frequently (AOR = 2.23, p < 0.001). The number of de facto household members was inversely correlated with folding or tying nets up (AOR = 0.92, p < 0.05), while respondents who had heard/seen a malaria message recently were 43% more likely to report practicing this net-care behaviour (p < 0.05). Lastly, the odds of folding or tying up a net was 78% lower in the Far North compared to respondents in the North region (AOR = 0.22, p < 0.001). No other background or ideational variables were significantly correlated with the dependent variable. Finally, residual clustering at the household level showed about 66.0% of the variance in the behaviour was attributable to household-level factors. Clustering at the enumeration area (EA) level was about 16.3%.

**Table 3:** Results of multilevel logistic regression of folding or tying up bed nets on selected variables

Correlates	Cameroon		Côte d’Ivoire		Sierra Leone	
	AOR	95% CI	AOR	95% CI	AOR	95% CI
Individual characteristics						
Gender						
Male (RC†)	1.000	–	1.000		1.000	
Female	1.107	0.792–1.548	1.456**	1.121–1.890	1.851***	1.342–2.555
Age in years	1.002	0.986–1.018	0.993	0.981–1.006	1.002	0.988–1.016
Education level						
None (RC)	1.000	–	1.000		1.000	
Primary	1.941	0.678–1.305	1.330*	1.007–1.756	1.678**	1.237–2.278
Secondary or above	1.105	0.610–2.000	1.242	0.930–1.660	1.144	0.827–1.582
Exposure to malaria-related messages						
None (RC)	1.000		1.000		1.000	
At least one message	1.426*	1.042–1.952	1.197	0.946–1.514	1.157	0.861–1.557
Consistent ITN use						
No (RC)	1.000		1.000		1.000	
Yes	2.233***	1.557–3.202	4.575***	3.367–6.215	1.560**	1.173–2.075
Ideational variables						
Positive attitudes towards net care (RC = negative attitude)	1.983*	1.112–3.535	1.850*	1.144–2.991	1.667*	1.083–2.565
Positive attitude towards the use of bed nets (RC = negative attitude)	2.079***	1.443–2.996	1.780**	1.146–2.763	0.982	0.754–1.278
Perceived severity of malaria (RC = Did not perceive)	0.893	0.643–1.240	0.616***	0.487–0.778	0.974	0.766–1.239
Perceived susceptibility for malaria (RC = Did not perceive)	1.800**	1.147–2.823	1.918***	1.460–2.520	1.297^§^	0.994–1.691
In the last six months, talked to spouse/partner, friends, or relations about malaria	0.862	0.583–1.274	2.200***	1.701–2.844	0.988	0.775–1.261
Perceived response-efficacy of bed nets (RC = Did not perceive)	0.576***	0.411–0.808	1.528***	1.208–1.932	1.920***	1.471–2.506
Perceived self-efficacy for consistent use of bed nets (RC = Did not perceive)	2.166***	1.452–3.230	1.218	0.829–1.789	2.507***	1.822–3.448
Perceived consistent use of bed nets was a community norm (RC = Did not perceive)	2.017 ***	1.409–2.889	1.498**	1.164–1.928	1.367^§^	0.934–2.002
Household/Community Variables						
Number of de facto household residents	0.924*	0.856–0.998	1.001	0.943–1.062	1.000	0.953–1.048
Number of nets in the household	1.127§	0.978–1.300	1.172**	1.046–1.314	0.983	0.858–1.125
Household wealth quintile						
Lowest (RC)	1.000		1.000		1.000	
Second	1.155	0.713–1.871	1.797**	1.180–2.736	0.885	0.616–1.273
Middle	1.174	0.676–2.038	2.290**	1.438–3.647	0.848	0.581–1.238
Fourth	0.847	0.465–1.542	1.799*	1.089–2.982	1.141	0.747–1.742
Highest	0.507§	0.255–1.007	2.004*	1/157–3.470	1.215	0.737–2.004
Type of place of residence						
Rural (RC)	1.000		1.000		1.000	
Urban	1.084	0.592–1.983	0.557**	0.371–0.838	2.13**	1.237–3.668
Region						
North region (RC)	1.000		—		—	
Far North	0.220***	0.126–0.385				
Zone						
North (RC)	—		1.000		—	
Centre			0.499*	0.290–0.858		
South			0.871	0.511–1.486		
Abidjan			0.616	0.314–1.208		
District						
Bo (RC)	—		—		1.000	
Port Loko					0.503***	0.342–0.739
Random Effects						
Variance: Household	4.806	3.481–6.636	9.378	7.377–11.269	0.793	0.431–1.458
ICC^‡^: Household	0.660	0.589–0.724	0.759	0.711–0.791	0.272	0.189–0.374
Variance: Cluster	1.571	1.002–2.464	0.999	0.636–1.565	0.435	0.247–0.768
ICC: Cluster	0.163	0.113–0.227	0.074	0.050–0.109	0.096	0.057–0.157
Number of observations	2940		6040		2730	

^§^p < 0.1; * p < 0.05; ** p < 0.01; *** p < 0.001

^†^Reference Category (RC); ‡ Intra-class Cluster Coefficient (ICC)

Survey data was collected during the rainy season between 2018 and 2019

### Côte d’Ivoire

#### Prevalence and variations in net-care behaviour

Among the 6,164 respondents from households with at least one ITN, about half (52.1%) reported folding or tying up a net when not in use as a way of minimizing tears and holes in nets. The reported prevalence of the behaviour was not different for men (51.3%) and women (52.4%) or by household wealth quintile, but there was a significant difference by place of residence with 48.7% of urban residents compared to 56.7% of their rural peers (p < 0.01). The behaviour also varied by zone; it was more common in the North (55.0%) and South (56.9%) zones compared to Abidjan (45.1%) and Central (50.4%) zones.

#### Multivariable analysis

Results of the multivariable, multilevel logistic regression model (Table 3) revealed several sociodemographic, ideational, household, and community variables that were significantly associated with net care. Consistent use of bed nets was the strongest correlate of the assessed net-care behaviour (AOR = 4.58; p < 0.001). Whereas age and exposure to malaria messages in the past year were not significantly associated with the assessed net-care behaviour, women were 46% more likely than men to report tying or folding up their nets (AOR = 1.46, p < 0.001). Seven of the eight ideational variables included in the estimated model were significantly associated with the outcome behaviour. The strongest positive ideational correlates were interpersonal communication about malaria, perceived susceptibility to malaria, perceived efficacy of ITN, descriptive norm about ITN use, positive attitudes towards ITN care, and positive attitudes towards ITNs. Discussing malaria with a friend or family member, positive attitudes towards net care more than doubled the odds of reporting net-care behaviour while positive attitudes towards net care increased the odds by 85%. Perceived susceptibility to malaria and positive attitude towards ITN use increased the odds of reporting the behaviour by 92 and 78%, respectively (AOR = 1.92, p < 0.001; AOR = 1.78, p < 0.01). Counter-intuitively, the association with perceived severity of malaria was negative (AOR = 0.62, p < 0.001).

Regarding household and community variables, an interesting pattern of relationships emerges. Every unit increase in the number of ITNs in the household is associated with a 17% increase in the odds of reporting the behaviour (p < 0.001). The relationship with wealth quintiles was such that the odds of reporting the behaviour were higher in the higher wealth quintiles than in the lowest wealth quintile. Furthermore, the respondents in the middle wealth quintile appeared to have the greatest odds of reporting the behaviours (AOR = 2.29, p < 0.001). Urban residents were 44% less likely to report the behaviour compared to their rural peers (p < 0.01). Differences by zone of residence were only significant when one compares the residents of the North zone with their counterparts from the Centre (AOR = 0.49; p < 0.05). Finally, the data revealed significant residual clustering at the household level with about 75.5% of the variance in the behaviour attributable to household-level factors. Clustering at the EA level was less pronounced but nonetheless significant.

### Sierra Leone

#### Prevalence and variations in net-care behaviour

Of the 2,730 respondents from households with at least one ITN, more than three-quarters (77.1%) reported that they had slept under an ITN every night, and 76.5% reported that they fold or tie up a net when it is not in use to minimize occurrence of tears and holes. The prevalence of this behaviour varied significantly by gender and by district. Specifically, a higher proportion of females (77.7%) than males (70.2%) (p < 0.001), and substantially more from Bo district (82.7%) compared to Port Loko district (71.2%) reported this behaviour (p < 0.001). Net-care behaviour also varied by residence and education level with a higher proportion of respondents from urban (85.3%) compared to rural (74.9%) areas (p < 0.001), and those with primary level of education (80.6%) compared to none (72.8%) or secondary and higher (80%), performing the behaviour (p < 0.001). The reported prevalence of the behaviour did not vary by wealth quintile.

#### Multivariable analysis

The adjusted, multivariable, multilevel logistic regression showed that several socio-demographic and ideational variables were significantly correlated with appropriate care of a net when it was not in use. Consistent use of bed nets was significantly correlated with net-care behaviour (AOR = 1.56, p < 0.01). District, residence, gender, and level of education also were significant predictors of net care. Respondents from Port Loko district were 50% less likely to perform net-care behaviour compared to those from Bo district (p < 0.001), and those residing in urban areas were more than two times more likely (AOR = 2.13, p < 0.01) than their rural counterparts to do so. Females were 85% more likely to perform net care compared to males (p < 0.001), and respondents with only a primary level of education were 68% more likely to perform appropriate net care compared to those with no education (p = 0.01). Age and exposure to malaria-related messages were not associated with the behaviour.

The odds of performing effective net care were elevated among respondents who had positive attitudes towards net care, were confident in their ability to use nets consistently, and who perceived nets to be effective protection against malaria. The odds of net care among respondents with positive attitudes towards net care were 1.7 times higher than similar respondents without positive attitudes (AOR = 1.67, p < 0.05). The odds of performing net care were almost doubled among respondents with perceived response efficacy of bed nets (AOR = 1.92, p < 0.001) compared to those without response efficacy. Similar results were found for respondents with self-efficacy to use nets consistently (AOR = 2.51, p < 0.001) compared to those without self-efficacy. Positive attitudes towards use of bed nets, perceived severity of malaria, discussing malaria with a friend or family member, and perception that net care was a community norm were not associated with performing the net-care behaviours. Perceived susceptibility to malaria (AOR = 1.30, p = 0.055) and perception that net use was a community norm (AOR = 1.37, p = 0.10) were marginally associated with net-care behaviour (see Table 3).

Finally, there was some residual clustering at the household level with about 27.2% of the variance in the behaviour attributable to household-level factors. Clustering at the EA level was less pronounced at about 10%.

## Discussion

This paper used data from Cameroon, Côte d’Ivoire and Sierra Leone collected between 2018 and 2019 to compare ideational, sociodemographic and household/community factors associated with care of ITNs when they are not in use. The specific net-care behaviour that was examined was tying or folding a hanging ITN up in order to keep the net away from factors that may cause damage to the net when it was not in use. The results revealed predictive factors that were common to the three countries and others that were country specific. These study results notably support limited previous studies on behavioural determinants of net care and provide additional understandings and implications for behaviour change programmes and research.

### Background variables

Respondents who used a net consistently were significantly more likely to tie or fold up an ITN in all three countries. The association between the two behaviours was strongest in Côte d’Ivoire (4.5-fold increase in odds of the net-care behaviour compared to those not using their net consistently) and least strong in Sierra Leone at 1.5 times greater odds. Net care is an important aspect of net use as to ensure longevity of the nets in use in the home, individuals must simultaneously care for the nets as they use them [36]. This is also consistent with the finding that damaged nets are more likely to be discarded, repurposed or no longer used [11]. No other literature with similar findings was found, however it appears logical that an individual using a net consistently may be more invested in taking care of their net, possibly out of concern for its ability to offer protection from mosquitoes and malaria. Timing of net distribution campaigns (which may provide information about caring for the new net) and administration of the survey in each of the countries may also have influenced net-care behaviours.

There were geographic differences in tying or folding ITNs up by region, zone, district, and urban/rural residence depending on the country. Living in the North region of Cameroon or Southern zone of Côte d'Ivoire was positively associated with tying and folding ITNs up. In Cameroon, the difference may be linked to the smaller household size in the North region. Study results indicate that average household size was negatively correlated with tying or folding up nets in Cameroon and household size is greater in the Far North region relative to the North Region. A second factor that may explain regional differences of the outcome in Cameroon is the timing of ITN distribution. While attitudes toward net care were similar in both Cameroonian regions, data collection in the Northern region coincided with ITN distribution activities. However, data collection in the Far North preceded ITN distribution. Residents in the Far North may have been less likely than those in the North to tie and fold up nets given the impending arrival of brand new ITNs in their area. In Sierra Leone, living in Bo district was positively associated with tying and folding ITNs up. The reasons for these geographic differences are unclear.

After adjusting for household wealth quintile and education level, results showed that respondents who lived in urban areas were more likely to tie or fold up nets in Sierra Leone only. No published work that explained similar findings was found.

None of the remaining sociodemographic variables (e.g., gender, education level) assessed were consistently associated with the outcome in all three countries. For example, being female was positively associated with tying and folding ITNs up in Sierra Leone and Côte d’Ivoire but not in Cameroon, and education level was associated with the behaviour in Côte d’Ivoire and Sierra Leone, while age was not associated with the behaviour in any of the countries. The association between gender and tying or folding ITNs up was stronger in Sierra Leone than in Cameroon and Côte d’Ivoire, and results showed that females were more likely than males to report practicing net-care behaviour. This finding is consistent with qualitative study results from Tanzania and Senegal that showed women were primarily responsible for making sure nets were maintained [31, 34]. In contrast, a quantitative study in Kenya [21] showed that nets used by females over the age of 50 years were not as well cared for as nets used by others in the household.

Attaining primary level education was significantly correlated with reported net-care behaviour in Sierra Leone and Côte d'Ivoire, but not in Cameroon. Having higher levels of education beyond the primary level was not significantly correlated with increased odds of net-care behaviour. The reasons for this finding are not clear. It is possible that adult respondents delegate net care to other less educated members of the household. Household characteristics varied in their prediction of reported net-care behaviours, and no household characteristic had a significant association with net-care behaviour across all three study countries. In Cameroon, the number of de facto household members was associated with decreased odds in reported folding or tying up of nets. The authors could not find published studies that explored the relationship between household size and practice of net care. Qualitative studies have found that some individuals consider net care time consuming and not a priority relative to other necessary day-to-day activities [32]. In Côte d’Ivoire, results showed that the number of nets in the household was significantly associated with increased net-care behaviours. The reason for this is not clear.

### Exposure to malaria messages

Exposure to malaria-related messages was linked to reporting appropriately caring for a net in Cameroon, but not in Sierra Leone or Côte d’Ivoire. There is a dearth of studies assessing the link between exposure to malaria-related messages and net-care behaviours. Koenker et al.’s study in Nigeria showed a correlation between exposure to behaviour change communication messages and increased positive attitudes towards net care and repair; exposure was also a strong predictor of net condition [20]. In addition, in the Koenker et al. study, the strength of the association increased with exposure to increasing numbers of communication channels. While all three countries reported implementing various SBC activities to promote malaria prevention and treatment in the months preceding the survey, including mass media and community mobilization interventions, the focus of this SBC on behaviours around net care was not documented.

### Ideational factors

Consistent with other studies that have examined the role of ideation on malaria-related behaviours [21, 27, 28], this study revealed the significant role that ideational variables play in influencing ITN-care behaviours. The study found significant association between seven ideational factors with reported net-care behaviour in at least two countries: positive attitudes towards net care and towards use of bed nets, perceived susceptibility to malaria, perceived response-efficacy of bed nets, perceived self-efficacy for consistent net use, and perceived norms about consistent net use in the community.

There was a significant correlation between a positive attitude towards net care and reported net-care behaviour in all the three countries. This finding is consistent with studies in Nigeria [20] and eastern Uganda [23] that showed that positive attitudes towards both net care and net repair were significantly associated with better condition of ITNs. Similarly, positive attitudes towards *use* of ITNs predicted net-care behaviours in both Cameroon and Côte d’Ivoire, but not in Sierra Leone.

Perceived susceptibility to malaria was strongly associated with reported net-care behaviour in Cameroon and Côte d’Ivoire, and less strongly associated with the outcome in Sierra Leone. Qualitative studies from Tanzania and Nigeria have found that risk perception and desire to protect self and family from mosquito bites and malaria were among the main motivating factors for practicing net-care behaviours [30, 32].

In Côte d’Ivoire, perceived severity of malaria was negatively associated with reporting tying or folding nets up; the reason for this relationship is unclear. The authors could not find published information on the relationship between perceived severity of malaria and net care. This finding contrasts with related results from qualitative studies [32, 34] that show some individuals believe correct net care offers protection from mosquitoes and serious illness.

This study showed that perceived self-efficacy to consistently use bed nets was associated with a more than two-fold increase in the odds of reported net-care behaviour in two countries. Few studies have explored the influence of perceived self-efficacy to practice net care on actual net-care behaviour although Santos et al. found that households where individuals perceived the self-efficacy to *prevent malaria* were less likely to correctly store their bed nets when they were not in use [21].

The results for perceived response-efficacy of bed nets were varied, yielding a positive association in Côte d’Ivoire and Sierra Leone, and a negative association in Cameroon. Limited information is available on the relationship between perceived effectiveness of nets and net-care behaviours, however a qualitative study in Senegal showed that participants equated effectiveness of their ITNs with good net-care behaviours including storing, washing and drying of nets [31].

Perceiving that consistent net use was a community norm was a strong predictor of net-care behaviours in Cameroon and Côte d’Ivoire, but not in Sierra Leone. No published information is available to explain this finding.

### Implications

The findings from this study have implications for programming, strategy and future research. Programme efforts to extend the average effective lifespan of ITNs should include SBC activities that prioritize messages about tying and/or folding ITNs up when they are not in use. While this study identifies several possible key ideational factors, observed differences in ideational correlates of net-care behaviours across countries reinforce the importance of tailoring country-specific SBC strategies to local context. SBC strategies that are informed by local formative evidence are more likely to resonate strongly with and be accepted by the population. Evidence from this study suggests that efforts to strengthen perceived response-efficacy of nets, perceived susceptibility to malaria, perceived self-efficacy for use of bed nets, positive attitudes towards proper net care, and the perception of net use as a community norm are relevant in the three study countries. National malaria SBC strategies that rely on maintaining high levels of ITN coverage and use should include promoting this net-care behaviour, as it has been shown to both extend effective ITN lifespan and increase ITN use. A balance of complementary behaviour change messages that promote consistent use of nets and reinforce tying and folding nets up when they are not in use during the day may be beneficial to durability of ITNs. The positive associations between being female and the assessed behaviour in Côte d'Ivoire and Sierra Leone suggests that women may take on this role, or there is a need for special efforts to reach men with relevant messages in these countries. Lastly, significant residual clustering at the household and community (cluster) levels indicates important unmeasured related factors operating at these micro levels.

## Limitations

This study has some limitations that warrant mention. First, the data used in the analysis are cross-sectional, precluding causal inferences, and the results presented are associations. Nonetheless, the strength of the associations (both in effect size and statistical significance) indicates that the results have relevance for programmatic decision-making. Second, the data describe only households with at least one ITN and only two sub-national areas within Cameroon and Sierra Leone, so findings may not be generalizable beyond these areas. Second, the variables in the analyses are based on self-report, thereby making the responses subject to social desirability bias. Given the precautionary measures taken during field work to ensure that interviewees provide objective responses, such as survey questions with unprompted responses, the risk for social desirability is possibly minimized.

## Conclusions

A study of the ideation factors associated with tying or folding up a bed net conducted in Côte d’Ivoire, Cameroon and Sierra Leone identified several psychosocial variables with potential to influence recommended net-care practices. SBC activities fostering increased consistent use of nets, positive attitudes towards net care, increasing perceived susceptibility of malaria infection, increasing perceived self- and response-efficacy of bed nets, and establishing perceived use of bed nets as a community norm may increase net-care behaviour.

## Supplementary Information

**Additional file 1: Table A1**. Question and statements used to measure ideational independent variables. Table describes the questions and statements used to create binary independent ideational variables used in the paper’s multilevel analysis.

## Data Availability

The Malaria Behavior Survey datasets are available through application to the USAID open access Development Data Library at https://data.usaid.gov.

## Abbreviations

ANC: Antenatal care
AOR: Adjusted odds ratio
EA: Enumeration area
ICC: Intra-class Cluster Coefficient
ITN: Insecticide-treated mosquito net
IPTp-SP: Intermittent preventive treatment in pregnancy with sulfadoxine–pyrimethamine
IPTi: Intermittent preventive treatment in infants
IRS: Indoor residual spraying
MBS: Malaria Behavior Survey
MIS: Malaria Indicator Survey
MICS: Multiple Indicator Cluster Survey
PMI: US President’s Malaria Initiative
RC: Reference category
SBC: Social and behaviour change
USAID: United States Agency for International Development
WHO: World Health Organization
