# Supplementary material for: Ideational factors associated with net care behaviour: a multi-country analysis
Source: Malar J. 2022 Feb 17;21:53. doi: 10.1186/s12936-022-04053-5 (PMC8851768; doi:10.1186/s12936-022-04053-5)
Supplement: Supplementary file 1 — Additional file 1: Table A1. Question and statements used to measure ideational independent variables. Table describes the questions and statements used to create binary independent ideational variables used in the paper’s multilevel analysis. [file 12936_2022_4053_MOESM1_ESM.docx]

# Additional file information

**File format:** Microsoft Word table

**Title:** Table A1 Question and statements used to measure ideational independent variables

**Description:** Table describes the questions and statements used to create binary independent ideational variables used in the paper’s multilevel analysis.

| **Additional file 1 Table A1 Question and statements used to measure ideational independent variables** | |
| --- | --- |
| Variable | Question/Likert statement |
| Positive net care attitudes* | There are steps/actions a person could take to make bed nets last longer |
|  | A person could protect his/her family from malaria by taking good care of the nets in the household |
| Positive attitudes towards net use* | It is easier to get a good night’s sleep when I sleep under a mosquito net |
|  | I do not like sleeping under a mosquito net when the weather is too warm |
|  | The smell of the insecticide makes it uncomfortable to sleep under a mosquito net |
|  | Mosquito nets are generally easy to use |
|  | More expensive mosquito nets are more effective than less expensive or free mosquito nets (for sleeping) |
|  | Insecticide-treated nets are safe to sleep under/ Insecticide-treated nets do not pose a risk to one’s health |
| Perceived severity of malaria * | You don’t worry about malaria because it can be easily treated |
|  | Only weak children can die from malaria |
|  | Every case of malaria can potentially lead to death |
|  | When someone you know gets malaria, you usually expect them to completely recover in a few days |
| Perceived susceptibility to malaria* | People in this community only get malaria during rainy season |
|  | Nearly every year, someone in this community gets a serious case of malaria |
|  | When your child has a fever, you almost always worry that it might be malaria |
|  | During the rainy season, you worry almost every day that someone in your family will get malaria |
| Discussed malaria with spouse, friends, or relations in the last six months | In the last six months, did you talk about malaria with your spouse or partner?  In the last six months, did you talk about malaria with your friends or relations? |
| Perceived response efficacy of nets* | Mosquito nets only prevent mosquito bites when used on a bed |
|  | My chances of getting malaria are the same whether or not I sleep under a mosquito net |
|  | Sleeping under a mosquito net every night is the best way to avoid getting malaria |
| Perceived self-efficacy for net use* | Sleep under a mosquito net for the entire night when there are lots of mosquitoes |
|  | Sleep under a mosquito net for the entire night when there are few mosquitoes |
|  | Sleep under a mosquito net every night of the year |
|  | Get all of your children to sleep under a mosquito net every night of the year |
| Perceived net use as the norm in one’s community | Generally, among the people in your community, how many sleep under them every night? Would you say…   - All people - Most people - At least half of the people - Fewer than half of the people - Don’t know |
| * Variable was created based on scoring and dichotomizing the sum of scored Likert scale questions. | |
